# Cationic peptide carriers enable long-term delivery of insulin-like growth factor-1 to suppress osteoarthritis-induced matrix degradation

**DOI:** 10.1186/s13075-022-02855-1

**Published:** 2022-07-20

**Authors:** Armin Vedadghavami, Bill Hakim, Tengfei He, Ambika G. Bajpayee

**Affiliations:** 1 Department of Bioengineering, Northeastern University, Boston, MA USA; 2 Departments of Mechanical Engineering, Northeastern University, Boston, MA USA

**Keywords:** Insulin-like growth factor-1, Charge-based drug delivery, Intra-cartilage depot, Cationic peptide carriers, Electrostatic interactions, Cartilage therapy

## Abstract

**Background:**

Insulin-like growth factor-1 (IGF-1) has the potential to be used for osteoarthritis (OA) treatment but has not been evaluated in clinics yet owing to toxicity concerns. It suffers from short intra-joint residence time and a lack of cartilage targeting following its intra-articular administration. Here, we synthesize an electrically charged cationic formulation of IGF-1 by using a short-length arginine-rich, hydrophilic cationic peptide carrier (CPC) with a net charge of +14, designed for rapid and high uptake and retention in both healthy and arthritic cartilage.

**Methods:**

IGF-1 was conjugated to CPC by using a site-specific sulfhydryl reaction via a bifunctional linker. Intra-cartilage depth of penetration and retention of CPC-IGF-1 was compared with the unmodified IGF-1. The therapeutic effectiveness of a single dose of CPC-IGF-1 was compared with free IGF-1 in an IL-1α-challenged cartilage explant culture post-traumatic OA model.

**Results:**

CPC-IGF-1 rapidly penetrated through the full thickness of cartilage creating a drug depot owing to electrostatic interactions with negatively charged aggrecan-glycosaminoglycans (GAGs). CPC-IGF-1 remained bound within the tissue while unmodified IGF-1 cleared out. Treatment with a single dose of CPC-IGF-1 effectively suppressed IL-1α-induced GAG loss and nitrite release and rescued cell metabolism and viability throughout the 16-day culture period, while free IGF at the equivalent dose was not effective.

**Conclusions:**

CPC-mediated depot delivery of IGF-1 protected cartilage by suppressing cytokine-induced catabolism with only a single dose. CPC is a versatile cationic motif that can be used for intra-cartilage delivery of other similar-sized drugs.

## Introduction

Osteoarthritis (OA) is a debilitating joint disease affecting more than 250 million people worldwide [1]. It is a major cause of pain and disability in adults yet remains without a cure [2]. OA onset results in the upregulation of several inflammatory cytokines including interleukin 1 (IL-1) and tumor necrosis factor-alpha (TNF-α) which in turn upregulate the expression of proteolytic enzymes that break down the cartilage matrix [3, 4]. Although there are potential disease-modifying OA agents that have been shown to inhibit this catabolic activity in preclinical studies, none have been successful in clinical studies, in part due to a lack of effective drug delivery systems [5]. Because of its localized nature, intra-articular (IA) administration is used as the primary route for the delivery of therapeutics to the individual affected joint. However, most of the administered drug is rapidly cleared out through the lymphatics and synovium vasculature preventing them from reaching their cell and matrix target sites in cartilage [5]. Additionally, the dense avascular matrix of cartilage is made up of a network of collagen II enmeshed with densely packed aggrecans comprising a high density of negatively charged sulfated glycosaminoglycans (GAGs), which hinders the passive diffusion of drugs in cartilage [5]. Consequently, developing effective cartilage-targeting drug delivery strategies that can enable intra-cartilage drug depots for sustained therapeutic doses over prolonged periods is necessary [6].

**Fig. 1:**
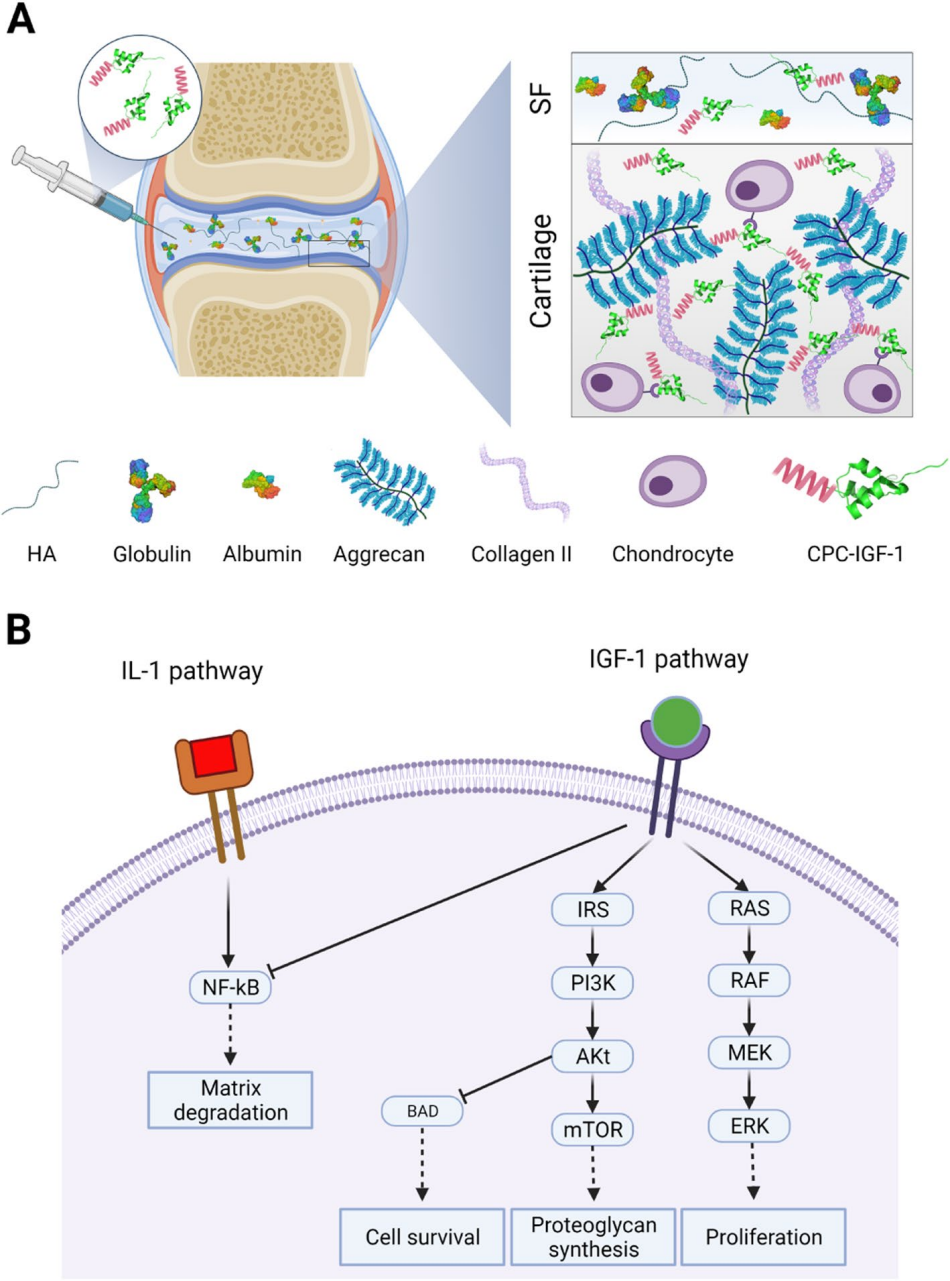
The concept of charge-based targeting of cartilage by using cationic peptide carrier (CPC) to deliver a pro-anabolic growth factor, IGF-1. **A** CPC +14N is comprised of 14 positively charged arginine (R) residues symmetrically distributed along the peptide length with hydrophilic asparagine (N) used as spacers. Its hydrophilic property minimizes its competitive binding within the synovial fluid. CPC-IGF-1 can rapidly penetrate through the full thickness of cartilage creating a drug depot to provide therapeutic drug doses over several days. **B** IGF-1 promotes chondrocyte proliferation, proteoglycan synthesis, and cell survival by activating the IGF-1 receptor. IGF-1 also suppresses IL-1-induced catabolism by downregulating the NF-kB pathway

The high negative fixed charge density (FCD) of cartilage can be utilized to enhance the uptake and retention of drugs in cartilage by making them cationic [5, 7–12]. Recently, we developed short-length cartilage-targeting arginine-rich *C*ationic *P*eptide *C*arriers (CPCs) with the goal of delivering large-size protein drugs to cartilage and demonstrated that a net charge of +14 (a sequence containing 14 arginines (R) residues symmetrically distributed along the length of the polypeptide) resulted in greatest intra-cartilage uptake, rapid and full-tissue-thickness penetration owing to weak-reversible binding nature of electrostatic interactions, and long-term retention in both healthy and arthritic cartilage explants (Fig. 1A) [13]. We further optimized the sequence to include hydrophilic asparagine (N) spacers (CPC +14N, (RRRR(NNRRR)_3_R) to minimize its competitive binding within the synovial fluid (SF) and enhance its cartilage targeting property (Fig. 1A).

Insulin-like growth factor 1 (IGF-1, MW ~7.6 kDa) is a pro-anabolic growth factor that plays an important role in cartilage matrix metabolism and repair mediated via multiple anabolic pathways [14, 15]. Importantly, IGF-1 can promote proteoglycan synthesis and cell survival by activating the PI3K/Akt/mTOR pathway (Fig. 1B) [16]. IGF-1-mediated activation of Ras and Raf/MAPK/ERK (MEK) and downregulation of NF-kB are known to induce chondrocyte proliferation and inhibit OA-associated catabolism (Fig. 1B) [17–19]. While IGF-1’s effectiveness for OA treatment has been extensively confirmed in vitro [14], it has not been evaluated in clinical trials yet. Its systemic use is associated with off-target effects including increased risk for cancer [20]. In addition to short intra-joint residence time following local IA delivery and a lack of cartilage targeting, IGF-1 also suffers from competitive binding from its binding proteins (IGF-BPs) which are known to be elevated in OA and aging and may prevent interactions with its receptors [21, 22]. Therefore, delivery strategies that can enable effective cartilage targeting and prolonged therapeutic response with only a single dose IA administration of IGF-1 while minimizing binding with BPs are required. Herein, our goal is to use CPC +14N for intra-cartilage delivery of IGF-1 and demonstrate that CPC +14N possesses sufficient electrical driving force to carry IGF-1 into the deep zones of cartilage tissue and bind there with aggrecan-GAGs to create a drug depot.

Here, we synthesize a cationic version of IGF-1 by conjugating it to cartilage homing CPC +14N (CPC-IGF-1) and compare its intra-cartilage transport properties with unmodified (free) IGF-1. We investigate the effectiveness of a single dose administration of electrically charged CPC-IGF-1 with unmodified IGF-1 in suppressing IL-1α-induced catabolism and rescuing cell metabolic activity in a well-established cartilage explant culture model over 16 days. The work demonstrates that cationic IGF-1 can penetrate through the full thickness of cartilage and retain within to create a drug depot that can provide therapeutic doses over the entire culture duration and suppress cytokine-induced GAG loss, rescue cell metabolism, and viability, and reduce nitrite production with only a *one-time dose*. This effect was significantly greater than a single dose of unmodified IGF-1 and only a continuous dosing regimen with free IGF-1 was able to match the response from a single dose of CPC-IGF-1.

## Materials and methods

### Materials

Human recombinant IL-1α and human recombinant IGF-1 were purchased from PeproTech (Rocky Hill, NJ). Dulbecco’s modification of Eagle’s medium (DMEM) was purchased from Corning (Corning, NY). Trypsin-EDTA phenol red, HEPES buffer, non-essential amino acids (NEAA), and penicillin streptomycin antibiotic-antimycotic (PSA) were purchased from Gibco (Carlsbad, CA). Ascorbic acid (AA) and L-proline were from Fisher Bioreagents (Pittsburgh, PA). Proteinase-K was purchased from Roche Diagnostics (Risch-Rotkreuz, Switzerland). NHS-PEG_2-_Maleimide and Protease Inhibitor Mini Tablets were from Thermo Scientific Pierce (Rockford, IL). Propidium iodide (PI) was obtained from Thermofisher Acros Organics (Geel, Belgium). Tris-HCl buffer was purchased from Invitrogen (Grand Island, NY). Chondroitinase ABC, Resazurin sodium salt, Griess reagent, fluorescein diacetate (FDA), and other salts and reagents were purchased from Sigma (St. Louis, MO).

### Design and synthesis of cationic peptide carrier (CPC)

Cartilage-penetrating Cationic Peptide Carrier, CPC +14N (RRRR(NNRRR)_3_R; MW = 3466 Da), rich in cationic arginine (R) and polar asparagine (N) residues as spacers with a net charge of +14 was synthesized by Fmoc solid-phase peptide synthesis (MIT Biopolymers and Proteomics, MIT, Cambridge, MA). An additional Cysteine was added at its C-terminus for conjugation with IGF-1 using sulfhydryl chemistry. The carrier was purified by reverse-phase C18 HPLC (yielding >95% purity) and its mass was confirmed using matrix-assisted laser desorption/ionization time-of-flight (MALDI-TOF) mass spectrometry.

### Synthesis and characterization of CPC-IGF-1 conjugates

**Fig. 2:**
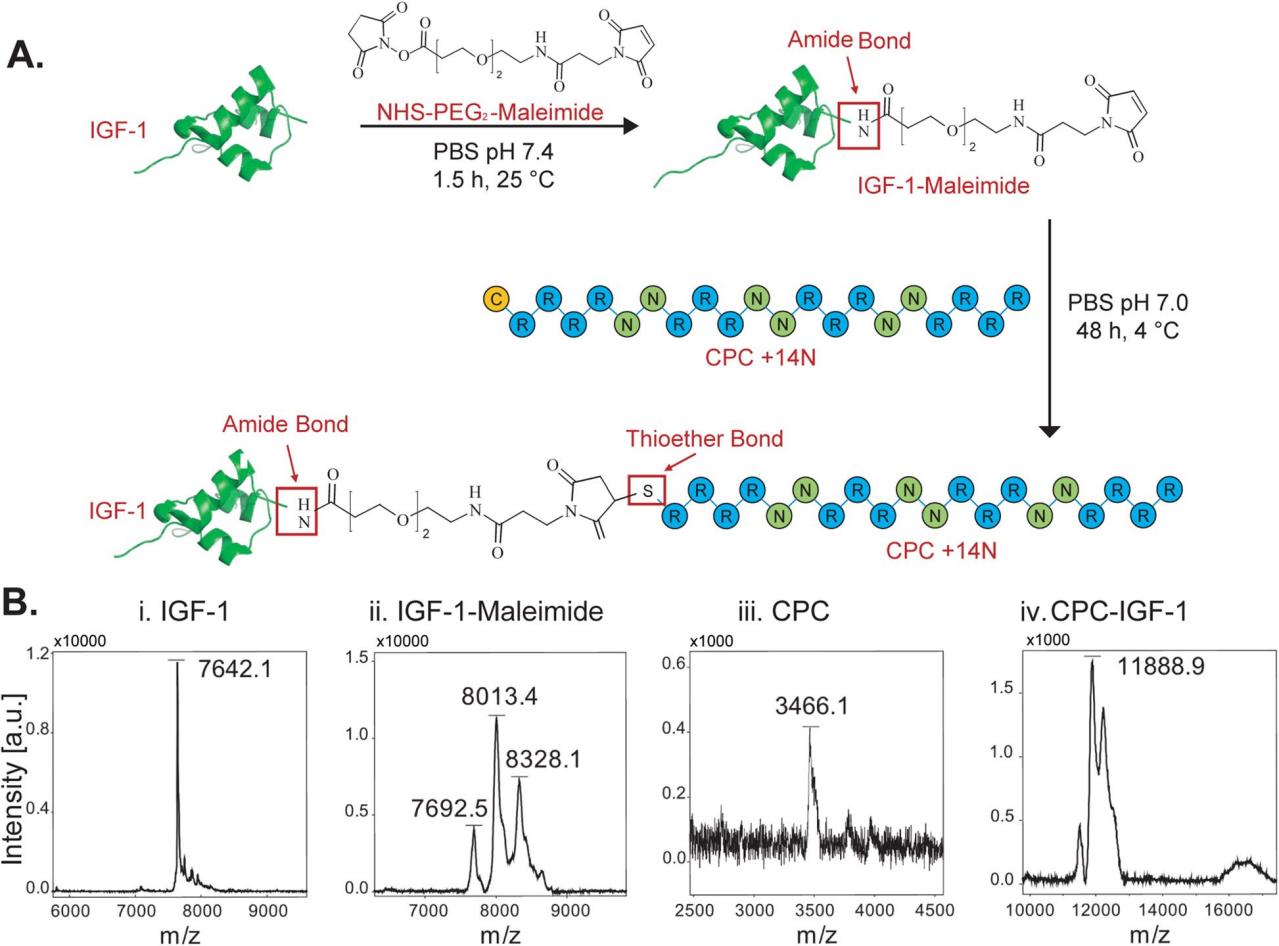
Scheme for the synthesis of CPC-IGF-1 formulation and MALDI mass spectrometry confirming conjugation of CPC to IGF-1. **A** IGF-1 was first modified with maleimide (IGF-1-Maleimide) through reaction with a bifunctional NHS-PEG_2_-Maleimide linker via targeting the primary amines on IGF-1, thereby adding the maleimide to IGF-1 through stable amide bonds**.** The maleimide-modified IGF-1 (IGF-1-Maleimide) was then reacted with CPC +14N consisting of a cysteine residue to form CPC-IGF-1 conjugate through a stable thioether bond. **B** MALDI mass spectrometry confirming conjugation of CPC to IGF-1

CPC +14N was conjugated to IGF-1 using a bifunctional NHS-PEG_2_-Maleimide linker. First, IGF-1 was reacted with NHS-PEG_2_-Maleimide to modify IGF-1 with maleimide through a reaction of the NHS group of the linker with the primary amines on IGF-1. Since there are no free sulfhydryl groups available in the IGF-1 structure, no cross-reaction between the maleimide group of the linker and IGF-1 cysteines occurs. Briefly, 50 μg (6.5 nmol, 1.0 equiv.) of IGF-1 in 50 μl of DI water was diluted in 200 μl of PBS pH 7.4 and reacted with 14 μg (32.9 nmol, 5.0 equiv.) of NHS-PEG_2_-Maleimide in 14 μl of DMSO for 90 min at room temperature (Fig. 2A). The reaction was quenched by spiking the reaction mixture with glycine. The reaction mixture was then subjected to two consecutive centrifugal filtration steps (3 kDa MWCO) at 8000g and 10000 g for 25 min each to remove free NHS-PEG_2_-Maleimide and exchange the buffer to PBS pH 7.0. The obtained IGF-1-Maleimide was reacted with CPC (31.7 nmol, 4.9 equiv.) for 48 h at 4 °C to synthesize CPC-IGF-1 conjugate.

Modification of IGF-1 with maleimide group (IGF-1-Maleimide) and the final CPC-IGF-1 conjugate was confirmed using Matrix-assisted laser desorption/ionization time-of-flight (MALDI-TOF) mass spectrometry (Bruker Microflex II). Briefly, 2 μL of IGF-1-Maleimide solution and CPC-IGF-1 (each at 0.16 mg/mL IGF-1 concentration) were mixed with equal volume of sinapic acid matrix (10 mg/mL). Changes in molecular weight of samples were detected and compared to that of CPC and IGF-1 stock solutions.

### Cartilage explant harvest

Cartilage plugs were harvested from the knee joints of 2-week-old bovine obtained from a local slaughterhouse (Research 87, Boylston, MA). Initially, full-thickness plugs were extracted from the femoropatellar grooves using 3 mm or 6 mm diameter biopsy punches. The plugs were then sliced to obtain 1 mm thick cartilage disks containing the superficial zone as described previously [23, 24]. Upon harvest, the disks were washed and equilibrated in PBS supplemented with PSA for up to 1 h. 3 mm explants were equilibrated individually in untreated control media comprised of DMEM supplemented with 1% HEPES buffer, 1% NEAA, 1% PSA, 0.4% L-proline, and 0.4% AA for 2 days at 37 °C and 5% CO_2_ prior to any treatment. The 6 mm explants were stored in PBS supplemented with proteinase inhibitors at −20 °C until the day of the experiment.

### Characterization of intra-cartilage transport properties

#### Equilibrium intra-cartilage uptake in normal and GAG depleted explants

**Fig. 3:**
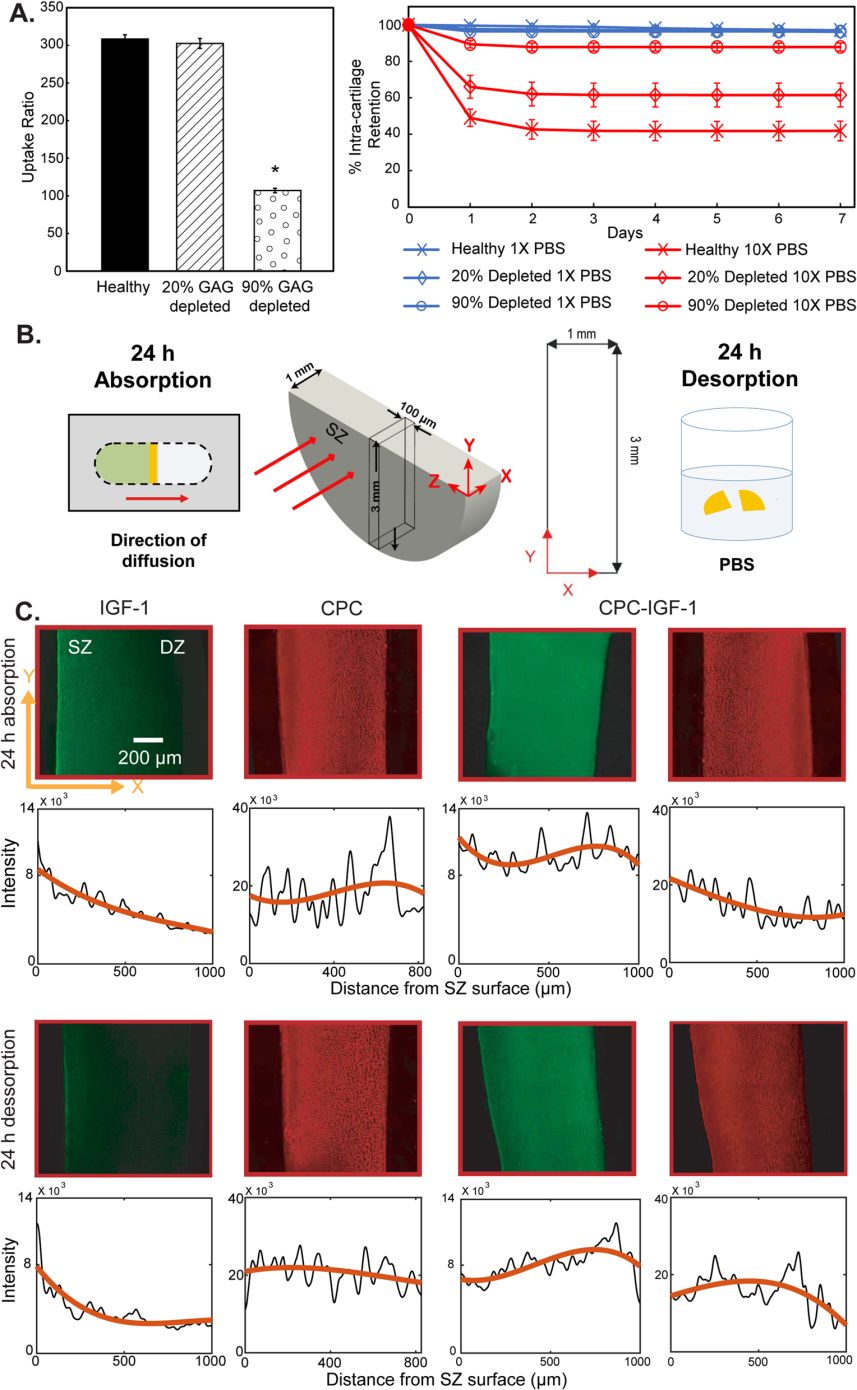
**A** Intra-cartilage uptake ratios of CPCs in healthy, 20%, and 90% GAG depleted cartilage explants (* vs healthy explant) and their % retention within cartilage over a 7-day desorption period in 1× and 10× PBS. **B** Half cartilage disks are placed in a custom-designed transport chamber. 1-D diffusion of fluorescent solutes is allowed through the cartilage explant from the superficial zone (SZ) to the deep zone (DZ) for 24 h. A 100 μm slice is cut from the center of the explant and imaged in the X-Y plane to estimate the depth of penetration of solutes from SZ to DZ. The equilibrated explants are also desorbed in 1× PBS for 24 h and imaged to estimate retention of solutes in cartilage. Adapted with permission from [7, 13]. **C** Confocal images showing 1-D depth of penetration of free IGF-1, CPC, and dual-labeled CPC-IGF-1 conjugate in cartilage after 24 h absorption followed by 24 h desorption in 1× PBS with their respective penetration concentration profiles. The green channel shows IGF-1-FITC and the red channel shows CPC-Cy5. SZ is the superficial zone and DZ and deep zone

Three ×1 mm normal cartilage disks were treated with 0.1 u/ml of Chondroitinase ABC in 1M Tris-HCI, pH 8.0 for 8 h to induce ~20% GAG depletion and 0.1 mg/ml of trypsin-EDTA phenol red in PBS for 14 h to induce ~90% GAG depletion, both at 37°C. After the digestion, explants were washed three times in PBS and equilibrated for 1 h in PBS supplemented with protease inhibitor to quench the digestion. GAG depletion percentages were measured using dimethyl-methylene blue (DMMB) assay [25]. Then, normal and GAG depleted cartilage disks were separately equilibrated in 300 μl of fluorescently labeled 30 μM CPC +14N in a 96 well plate at 37 °C for 24 h. The change in CPC fluorescence before and after equilibration was measured by a microplate reader (Synergy H1, Biotek). The standard curve to correlate CPC +14N concentration with its fluorescence activity showed to be linear. The uptake ratio was obtained by normalizing CPC +14N concentration inside the cartilage by the equilibration bath’s CPC concentration (Fig. 3A).

#### Intra-cartilage retention

The 24 h equilibrated explants were desorbed in 1× or 10× PBS supplemented with protease inhibitor for 7 days. The desorbed CPC concentration was measured using a microplate reader every day and the bath was replaced with new PBS. Intra-cartilage retention was defined as the percentage of the total CPC moles retained within the explants after desorption by the initial CPC moles after the 24 h equilibration.

#### Fluorescent labeling of IGF-1

IGF-1 was tagged with fluorescein isothiocyanate isomer 1 (FITC) before conjugating to CPC +14N. IGF-1 buffer solution was exchanged to 0.1 M sodium carbonate buffer, pH 9 prior to the labeling reaction. 50 μg (6.5 nmol, 1.0 equiv.) of IGF-1 in 100 μl of 0.1 M sodium carbonate buffer at pH 9 was reacted with 8 μg (20.4 nmol, 3.1 equiv.) of FITC in DMSO for 18 h at 4 °C. The excess FITC was removed from IGF-1-FITC using multiple centrifugation steps using 3 kDa filters and exchanging buffer with PBS pH 7.4. Fluorescently labeled CPC +14N (Cy5) was conjugated to FITC labeled IGF-1 under the same reaction conditions as described in Section 2.3. The dual fluorescently labeled CPC-IGF-1 was used for evaluating its depth of penetration into cartilage tissue as explained below.

#### Depth of penetration

A previously described custom-designed transport chamber was used to study the depth of penetration of dual labeled CPC-IGF-1 from the superficial (SZ) to the deep zone (DZ) of cartilage and compare that with FITC labeled IGF-1 and Cy5-labeled CPC (Fig. 3B) [13, 26]. Briefly, 6 mm half-disk cartilage explants were placed in the center of the chamber. The upstream compartment was filled with either fluorescently labeled CPC +14N, IGF-1, or dual labeled CPC-IGF-1 at the same starting concentrations while the downstream chamber was filled with PBS. The chamber was placed in a covered petri dish with DI water inside an incubator at 37 °C under gentle shaking. After 24 h, a 100 μm slice was cut from the center of the explant and imaged using a confocal microscope (Zeiss LSM 800). Cy5 (red channel) was excited 640 nm laser and FITC (green channel) was excited using a 488 nm laser. Z-stacks of images from both channels were obtained and maximum intensity along the z-axis was projected. The intensity across the thickness to represent the penetration concentration profile was plotted along a straight line from the superficial to deep zone using FIJI. Cartilage explants equilibrated with labeled IGF-1, CPC and CPC-IGF-1 were then desorbed in 1x PBS for 24 h and imaged using the same procedure described above.

### In vitro cartilage explant culture model of post-traumatic OA and treatment conditions

**Fig. 4:**
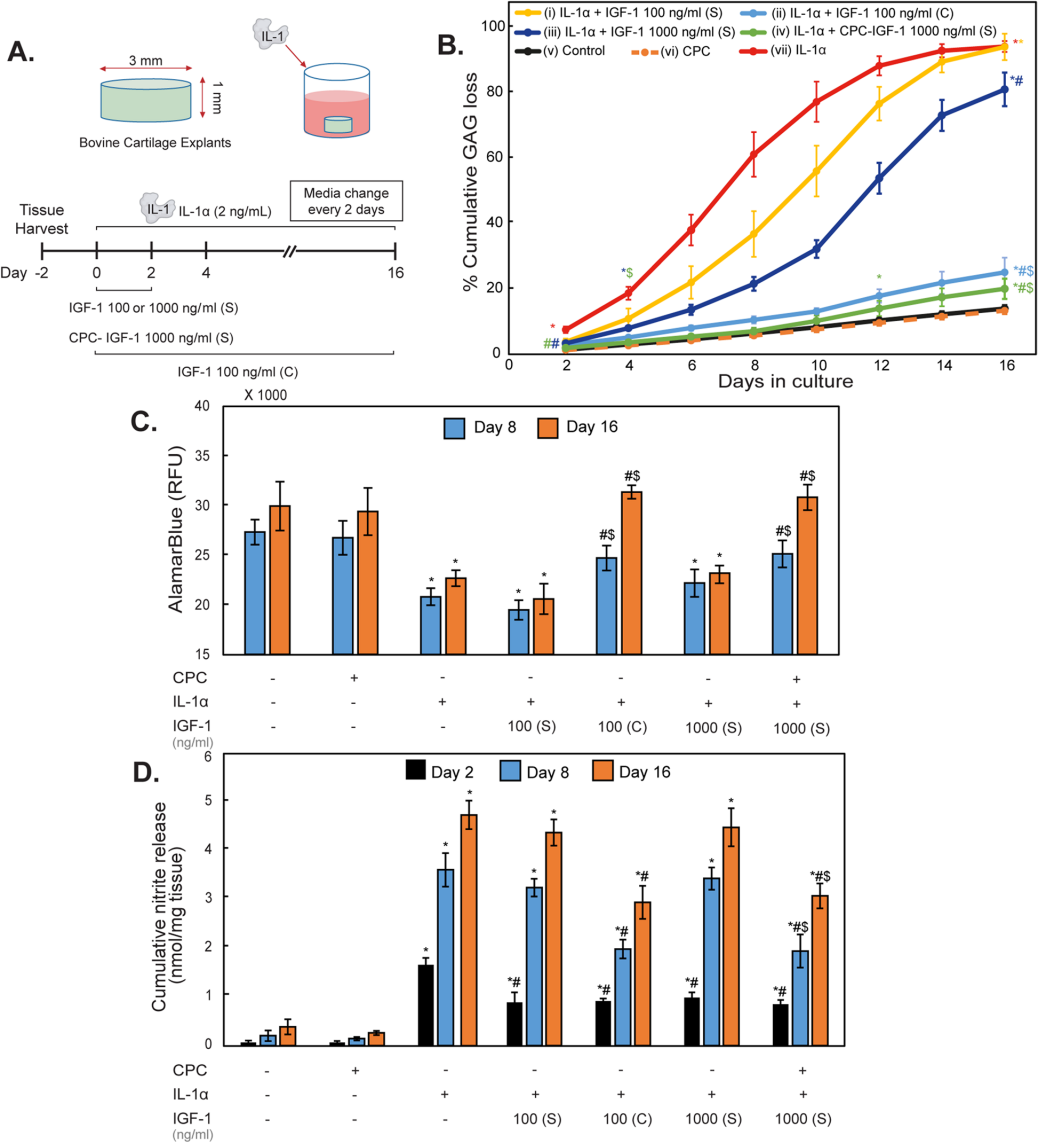
Experimental design to study biological effectiveness of CPC-IGF-1 using post-traumatic OA cartilage explant culture models. **A** Explants were treated with IL-1α (2 ng/ml) for the 16-day culture duration and received either (i) a single dose of 100 ng/ml (S) of free IGF-1, (ii) a continuous dose of 100 ng/ml (C) of free IGF-1, (iii) a single dose 1000 ng/ml (S) of free IGF-1, or (iv) a one-time dose of 1000 ng/ml (S) CPC-IGF-1 conjugate. (v) An untreated control, (vi) explants with single dose of CPC, and (vii) explants with IL-1α dosage only were also added as comparisons. **B** Percentage cumulative GAG loss to media over 16 days. **C** Cellular metabolism measured at days 8 and 16 using AlamarBlue assay. **D** Nitrite release determined by Griess assay at days 2, 8, and 16 (* vs control, # vs IL-1α, $ vs single dose 1000 ng/ml IGF-1 condition. In curve B, the statistical markers are color coordinated and all the data points enclosed within similar markers are statistically significant)

To compare the effect of a single (S) versus continuous (C) dose of IGF-1, cartilage explants were treated with 2 ng/mL of IL-1α for 16 days in combination with either (i) a single dose of IGF-1 at 100 ng/ml (S) or (ii) a continuous dose of IGF-1 at 100 ng/ml (C). Media was collected and changed every 2 days and IL-1α was replenished with each media change. In single-dose conditions, the cartilage explants were exposed to IGF-1 for only the first 2 days of culture, and further media changes did not contain IGF-1 to resemble the case of a single-dose IA injection in vivo [23, 27, 28]. However, for the continuous dose administration, IGF-1 was replenished in the culture with each media change. IGF-1 concentrations are based on work by Grodzinsky and colleagues which shows a dose-dependent IGF-1 response in suppressing IL-1 α-induced catabolic activity in a similar cartilage explant culture model with therapeutic effect saturating at a concentration of 100 ng/mL IGF-1 [22]. To evaluate the effect of one-dose intra-cartilage depot delivery, a 10x higher concentration of IGF-1 at 1000 ng/mL in CPC-IGF-1 was chosen to make therapeutic doses available to the chondrocytes for the entire culture duration of 16 days. As such, treatment groups with (iii) a single dose of free IGF-1 at 1000 ng/ml (S) and (iv) a single dose of CPC-IGF-1 conjugate containing IGF-1 at 1000 ng/ml (S) in combination with IL-1α were also included (Fig. 4A). As the baseline comparison, (v) an untreated control group without IL-1α and IGF-1, (vi) a condition treated with a single dose of CPC at the equivalent concentration as in the CPC-IGF-1 conjugate, and (vii) a group treated with only 2 ng/mL of IL-1α were also included in the study. IL-1α is chosen for this in-vitro post-traumatic (PTOA) model as it is elevated during the inflammatory phase following an injury and causes degradation of aggrecans, collagens, inhibits proteoglycan synthesis, and alters chondrocyte viability [29]. 2 ng/mL concentration was chosen as it represents a moderately aggressive cytokine treatment [22].

#### Sulfated glycosaminoglycan (GAG) loss from cartilage

After 16 days of culture, the cartilage explants were weighed and digested using Proteinase-K solution in Tris buffer at pH 8. The cumulative GAG released to the media and the residual GAG content in the digested tissue explants was measured using DMMB assay. The GAG content was expressed as a percentage of the total GAG content (in media + residual GAG in the digested explant).

#### Nitrite release from cartilage to medium

Nitrite content was measured using the Griess assay as a marker of nitric oxide (NO) release from cartilage into the medium. Equal volumes of Griess reagent and culture media collected every two days were mixed and incubated at room temperature for 15 min, and absorbance at 540 nm was measured using a microplate reader (Synergy H1, Biotek). Sodium nitrite solution in DMEM was used as a standard to convert absorbance readings to nitrite content in the sample medium.

#### Cellular metabolism in cartilage explants

On days 8 and 16 of culture, cellular metabolism in cartilage explants was determined by measuring the metabolic oxidation activity by using AlamarBlue assay [30]. Briefly, cartilage explants were individually incubated with media containing 1× resazurin sodium salt for 3 h in the dark at 37 °C and 5% CO_2_. Cell metabolic activity was estimated by measuring fluorescence at 530 nm excitation and 590 nm emission wavelengths using a microplate reader (Synergy H1, Biotek).

#### Chondrocyte viability in cartilage

On days 8 and 16 of culture, 100 μm thick slices were cut from the center of cartilage disks as described before [7]. Cartilage slices were stained with FDA (4 mg/ml in PBS) and PI (40 mg/ml in PBS) for 5 min. FDA stained viable cells green and PI stained the non-viable cells red. Slices were washed with PBS and imaged using a fluorescence microscope (Nikon Eclipse TS2R) at 4× objective. The viable and non-viable cell images were overlaid using ImageJ.

### Statistical analysis

All the data is presented as Mean ± Standard Deviation. For all studies, *n* = 8–10 explants were used per condition and experiments were repeated using explants from at least 3 animals yielding consistent results and trends. A general linear mixed-effects model with animal as a random variable was used. For comparisons between different treatment conditions, Tukey’s Honestly Significant Difference test was used. *P* < 0.05 was considered statistically significant.

## Results

### Confirmation and characterization of CPC-IGF-1 conjugate structure

MALDI-TOF confirmed the addition of 1 and 2 PEG_2_-Maleimide groups per IGF-1 as evidenced by the increase in molecular weight of IGF-1 from 7642.1 Da to 8013.4 and 8328.1 Da respectively (Fig. 2B i–iv). CPC to IGF-1 maleimide conjugation is indicated by the increase in molecular weight of IGF-1-Maleimide to 11,888.9 Da, showing incorporation of 1 mole of CPC (3466.1) per mole of IGF-1 (Fig. 2B iii–iv). The strong intensity of product peak at 11,888.9 Da shows a greater population of IGF-1-Maleimide in 1:2 molar ratio than 1:1, potentially due to the availability of a higher density of maleimide groups for binding with the CPC. Despite the presence of 2 maleimide groups on IGF-1, the final conjugation with CPC occurred only at 1:1 molar ratio.

### Intra-cartilage equilibrium uptake, retention, and depth of penetration of CPC +14N and CPC-IGF-1

High uptake of CPC +14N in the range of 300× was measured in both healthy and 20% GAG-depleted cartilage explants. However, when ~90% of GAGs were depleted, intra-cartilage uptake significantly dropped to 107.1 ± 1.9 (Fig. 3A) highlighting the dominant effects of electrostatic interactions. A majority of CPC molecules remained retained within healthy and GAG-depleted cartilage explants at least for 7 days (which is the longest period over which the desorption experiment was conducted). However, when desorbed in a high salt concentration bath (10× PBS), ionic interactions weakened resulting in a significant drop in CPC retention within two days (Fig. 3A) that plateaued at about 40–60% retention in healthy and 20% GAG depleted cartilage, and at about 90% in 90% GAG depleted tissue samples. Since all CPCs were not desorbed in 10× PBS, it indicates the presence of other types of short-range interactions like H-bonds that can stabilize intra-cartilage charge-based binding, as also reported in our previous works [13, 31].

Following 24 h absorption using a 1-D transport chamber (Fig. 3B), free IGF-1 penetrated through the full thickness of cartilage due to its relatively smaller size (7.6 kDa). However, a concentration gradient was evident along the tissue thickness showing higher concentrations of IGF-1 in the superficial zone (SZ) than in the middle and deep zones (DZ) (Fig. 3C). In contrast, the dual-labeled CPC-IGF-1 penetrated through the full tissue thickness (similar to CPC alone, red), and the conjugated IGF-1 (green) was present at a higher concentration throughout the cartilage thickness compared to free IGF-1 (Fig. 3C).

After 24 h of desorption in 1× PBS, most of the free IGF-1 desorbed out of the cartilage as IGF-1 binding was not strong enough to be retained within the tissue. In contrast, CPC-IGF-1 was retained through the full thickness of cartilage owing to electrostatic interactions between the optimally charged CPC and the negatively charged GAGs, confirming that CPC +14N provides sufficient electrical driving force to carry IGF-1 into the deep zones of cartilage and create a drug depot (Fig. 3C).

### A single dose of free IGF-1 is ineffective, and a continuous dose administration is necessary to suppress GAG loss, rescue cell metabolism and chondrocyte viability in IL-1α treated cartilage explants

The biological effectiveness of IGF-1 and CPC-IGF-1 was evaluated using 16-day cartilage explant culture models challenged with IL-1α as presented in Fig. 4A. IL-1α treatment resulted in increased breakdown of cartilage aggrecan sulfated GAGs that were released into the media over 16 days. Cumulative GAG loss to media was 6–7× higher in IL-1α treated explants compared to untreated control explants by day 16 (Fig. 4B). Initially, a single dose of free IGF-1 (100 ng/ml (S)) significantly inhibited GAG loss by day 2. However, this single dose failed to elicit a sustained response as GAG loss levels increased significantly through day 16 to become similar to the IL-1α condition (Fig. 4B). A continuous dose of 100 ng/ml IGF-1 was needed to keep the GAG loss levels down to control levels through the 16-day culture period (Fig. 4B). Increasing the dose to 1000 ng/ml for single administration of IGF-1 remained ineffective in providing a prolonged effect as 3–4× higher GAG loss levels compared to the continuous 100 ng/ml IGF-1 condition were measured by day 16.

**Fig. 5:**
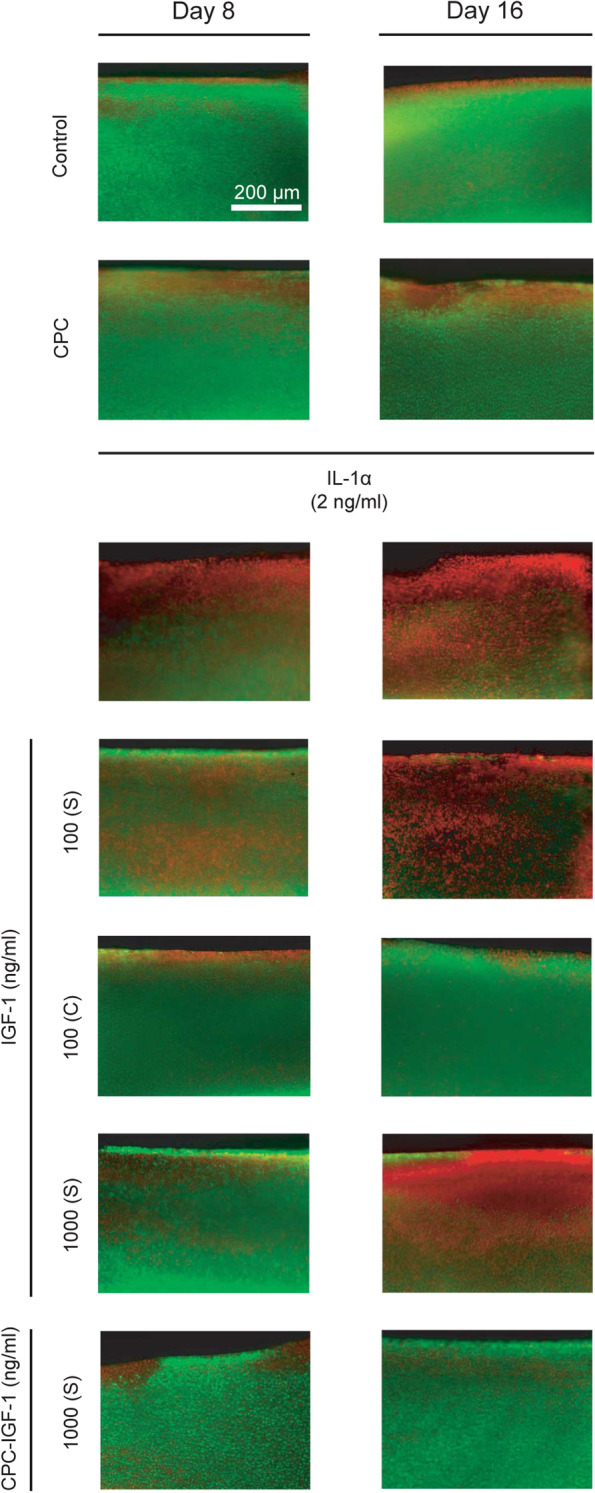
Chondrocyte viability after 8 and 16 days of culture. Viable cells are stained green while non-viable cells are marked red

As expected, treatment of cartilage explants with IL-1α resulted in a significant reduction of chondrocyte metabolic activity (measured using AlamarBlue) and viability (evaluated using FDA/PI live-dead staining) compared to untreated control as measured on days 8 and 16 (Fig. 4C and Fig. 5). A single dose of 100 ng/ml (S) of free IGF-1 was not effective in rescuing chondrocyte metabolic activity or viability and a continuous dose was needed to match the response from the untreated control condition. A single high dose of 1000 ng/ml (S) free IGF-1 also failed to rescue the reduced cell metabolism and viability over the long-term further reinforcing the importance of maintaining sustained therapeutic concentrations of the drug throughout the culture duration.

### A one-time dose of electrically charged CPC-IGF-1 effectively suppressed IL-1α-induced GAG loss, reduced nitrite release, and rescued chondrocyte metabolism and viability while a single dose of free IGF-1 did not

A one-time dose of cationic CPC-IGF-1 (1000 ng/mL, green curve in Fig. 4B) suppressed IL-1α-induced GAG loss (red curve, Fig. 4B significantly greater than the equivalent single dose of free (unmodified) IGF-1 (green curve) starting at day 4 and kept the levels down throughout the 16-day culture period similar to the control condition (black curve) and the continuous free low dose IGF-1 condition (100 ng/mL, C) (Fig. 4B), demonstrating that CPC-IGF-1 can form an intra-cartilage drug depot to provide sufficient therapeutic IGF-1 doses throughout the culture period. Similar trends were observed for chondrocyte metabolism rates. Compared to healthy control, chondrocyte metabolism rate was significantly reduced in IL-1α treated explants (Fig. 4C). While a single dose of free IGF-1 (1000 ng/mL) was ineffective in rescuing the reduced metabolic rate resulting from IL-1α treatment, a single equivalent dose of CPC-IGF-1 significantly increased the cell metabolic rate to that in healthy cartilage by both days 8 and 16 (Fig. 4C).

As expected, treatment with IL-1α resulted in the production of higher concentrations of nitrites on day 2 and greater cumulative nitrite production by days 8 and 16 compared to the control group (measured using Griess assay) (Fig. 4D). A single dose of free IGF-1 at a low dose of 100 ng/ml (S) or a high dose of 1000 ng/ml (S) suppressed nitrite production only on day 2 but was ineffective over the rest of the culture duration (Fig. 4D). In contrast, a continuous 100 ng/ml (C) dose of free IGF-1 significantly suppressed the IL-1-induced cumulative nitrite levels (Fig. 4D). Again, this response was matched by the single dose of CPC-IGF-1 that suppressed nitrite release throughout the 16-day period significantly greater than the one-time dose of free IGF-1 (Fig. 4D).

Similarly, cell death was rescued by a single dose of CPC-IGF-1 while the free IGF-1 condition was not effective (Fig. 5). Additionally, carrier control CPC alone condition at the equivalent concentration as in CPC-IGF-1 formulation was found to be safe as no changes in GAG loss (dashed orange curve, Fig. 4B), metabolic activity, nitrite release, or chondrocyte viability (Fig. 4C, D) were observed in comparison to the untreated control.

## Discussion

Here we design an optimally charged intra-cartilage depot forming cationic formulation of a 7.6-kDa IGF-1 protein for OA therapy. IGF-1 was conjugated to a short-length arginine-rich hydrophilic cationic peptide carrier with a net charge of +14 (CPC +14N) in a 1:1 molar ratio. CPC+14 was designed for greatest and full-thickness cartilage targeting resulting in 150–320× uptake in both healthy and arthritic cartilage with diminished FCD with long-term retention [13]. While SF has anionic hyaluronic acid and albumin, which may compete for binding with cationic solutes, our recent work shows that it is the hydrophobic (and not electric charge) interactions that are the dominant cause of binding within the SF [31]. The CPC design was thus modified to form CPC +14N, containing asparagine as spacers to make it hydrophilic. The carrier design is unique as the arginine facilitates the formation of non-electrostatic H-bonds with sulfated GAGs that synergistically stabilize long-range charge-based binding in cartilage [13, 31]. This provides CPC +14N a unique property to target and bind with arthritic cartilage where the negative FCD is reduced.

Our work shows that upon conjugation, CPC +14N possesses sufficient electrical driving force to carry IGF-1 into the deep pockets of cartilage tissue and enhance the drug’s intra-tissue retention thereby creating a drug depot. As a result, therapeutic doses of IGF-1 become available from this depot to its receptors on chondrocytes throughout the 16-day culture period with only a one-time dose that effectively suppressed cytokine-induced GAG loss, rescued cell metabolism and viability, and reduced nitrite release. In contrast, a single equivalent dose of free IGF-1 was not effective and only its continuous dosing regimen could enable a prolonged therapeutic response. A continuous dosing condition in this in vitro model is equivalent to multiple IA drug dose administration conditions, which is not a clinically viable option.

IGF-1 is a potent anabolic growth factor known to regulate proteoglycan biosynthesis and maintain chondrocyte and cartilage matrix homeostasis [15, 32, 33]. IGF-1 also has an anti-catabolic property; it has been shown to inhibit the NF- kB pathway in cytokine-challenged primary chondrocytes [19]. Similarly, IGF-1 administration decreased MMP-13 expression by 50% in endplate chondrocytes in part through activation of MAPK cascade [34]. With multiple dosing, it has been shown to significantly suppress GAG loss in IL-1 and TNF-α challenged cartilage culture models at a saturating concentration of 100 ng/ml [35, 36], which also motivated our continuous dosing condition. Therefore, exogenous administration of IGF-1 is a potential therapeutic for reversing OA progression owing to its capability in upregulating proteoglycan synthesis, increasing chondrocyte proliferation, enhancing cell survival, and downregulating OA-induced catabolism [14]. However, previous in vivo efforts using the intra-articular injection of free IGF-1 have been unsuccessful in part due to the short intra-joint half-life of IGF-1 [37, 38]; no retention of free IGF-1 was observed in cartilage even as early as 2 days post-IA injection in rat knees [37]. Similarly, using our in vitro transport model we showed that IGF-1 desorbed out of the cartilage within 24 h. Its intra-cartilage retention is expected to be even shorter in vivo owing to the rapid synovial fluid turnover rate and steric hindrance from the dense negatively charged cartilage matrix [5]. Therefore, approaches to localize IGF-1 in cartilage and enable prolonged availability are necessary for the successful translation of IGF-1 for OA treatment.

Here we utilized a simple aqueous-based, site-specific sulfhydryl chemistry for conjugating CPC +14N with a terminal cysteine to IGF-1 using an NHS-PEG_2_-maleimide linker, which mostly conjugates at the protein’s N terminal amine and is not expected to affect IGF-1’s binding to its receptors (IGF-1R). For example, Zhang et al incorporated an 8 amino acid epitope at the N-terminal of IGF-1 and found that the binding affinity of IGF-1 to IGF-1R was not altered [39]. In fact, the C domain of IGF-1 has been identified to have a high binding affinity to its receptors [40]. Since there are no primary amines present in the C domain (position 78–89: GYGSSSRRAPQTG), no modification occurs in this domain and thus the conjugation approach used here is not expected to interfere with its binding to chondrocyte receptors. It should be noted that guanidinium groups of arginine (pKa>12) remain protonated at the reaction buffer of pH 7.4 and cannot provide a nucleophilic site for reaction with NHS ester [41]. It has also been suggested that N terminal modification of IGF-1 significantly reduces binding with its BPs [42, 43]. Thus, conjugation of CPC to IGF-1 at its N terminal may be advantageous in increasing its bioavailability through reduced affinity towards IGF-1BPs. Further studies are needed to measure the binding affinity of CPC-IGF-1 with IGF-1BPs to confirm this phenomenon.

OA onset results in an initial increase in IGF-1 levels owing to activation of reparative processes, however, the rate of catabolic degradation prevails over the repair processes with OA progression resulting in significant IGF-1 loss and reduction of IGF-1 mRNA expression in cartilage [44]. Despite the initial rise in the synovial fluid concentration of IGF-1, elevated levels of IGF-BPs are also reported in some OA patients [45, 46]. IGF-1 binds with the IGF-BPs with a similar affinity to IGF-1R [47], suggesting that OA and aging chondrocytes respond poorly to IGF-1 in part due to increased levels of IGF-BPs in the matrix [21]. However, it has been shown that human OA culture explants remained responsive to exogeneous IGF-1 administration; while a higher dose of 30 ng/ml IGF-1 was required to induce 2.6-fold enhanced proteoglycan synthesis in advanced OA cartilage explants (MANKIN score 8), a low dose of 3 ng/ml IGF-1 was sufficient to increase proteoglycan synthesis by 1.8-fold in a milder degree of OA explants (MANKIN score 4) [48]. In 3D young bovine chondrocyte culture, exogenous IGF-1 enhanced expression levels of IGF-1 and IGF-1R but not of BPs [49]. Taken together, these results suggest that even though a lower therapeutic benefit is expected from IGF-1 in presence of its BPs, it should not result in its complete irresponsiveness. This also indicates the importance of administering IGF-1 during the early stage of OA for effective repair as its responsiveness and BP concentration are known to be influenced by disease severity [47]. This is consistent with the in vitro results showing significant suppression of OA-induced GAG loss using IGF-1 in the early stages of post-traumatic OA [22].

The high negative FCD of cartilage resulting from the negatively charged GAGs provides a unique opportunity to make drug cartilage penetrating using cationic carriers [5, 8, 50, 51]. A Fusion protein consisting of IGF-1 and a heparin-binding (HB) motif was developed that localized and retained IGF-1 in cartilage via binding with heparan sulfate GAGs [42, 52]. This heparin-binding motif is comprised of positively charged lysine and arginine residues sequenced to have a high binding affinity towards heparan sulfate (K_D_ of 21 nM) [42]. The heparin-binding fusion IGF-1 (HB-IGF-1) protein was retained in cartilage 24 h post-IA injection in rats while no free IGF-1 was detectable in any joint tissues at the same timepoint [42]. Additionally, in a rat medial meniscal tear OA model, surface and full-depth cartilage loss were significantly reduced by HB-IGF-1 compared to free IGF-1 due to its prolonged intra-cartilage retention [37]. Polyamidoamine (PAMAM) dendrimers are a group of hierarchically branched cationic nanoparticles (Gen 6, 58 kDa) that have also been tried for localizing IGF-1 in cartilage [43]. The PAMAM nanoparticle allowed prolonged bioavailability of IGF-1 for 30.4 days while free IGF-1 was only available for 2.9 days in the rat knee joint space post-IA injection [43]. In rat cartilage samples, PAMAM-IGF-1 was detected 6 days post-IA injection while no trace of free IGF-1 was detectable [43]. PAMAM-IGF-1 significantly suppressed matrix degradation and osteophyte formation compared to free IGF-1 post-IA injection in anterior cruciate ligament (ACL) transected (ACLT) rat knee joints [43]. These results validate using electrostatic interactions for targeted delivery of IGF-1 to cartilage and enabling a prolonged biological benefit. PAMAM dendrimers, however, have been shown to induce a generation-dependent cytotoxic response in mammalian cells through DNA damage and cell apoptosis [53]. For example, treatment of HaCaT cells with 3.2 μM of Gen 4, 5, and 6 PAMAM dendrimers resulted in 4.7%, 25.9%, and 89.6% DNA breakage, respectively [54]. PAMAM dendrimers have also been shown to induce mitochondria-mediated apoptosis following endocytosis through lysosomal pathways [55]. These severe cytotoxicity concerns limit its applications in drug delivery and its potential for clinical translation [56, 57]. CPCs, on the other hand, are short-length peptides containing natural L-amino acids shown to have no adverse effects on GAG loss, metabolism, nitrite release, and chondrocyte viability compared to untreated control at the equivalent dose (3.1 μM CPC +14N) used in single-dose of 1000 ng/mL CPC-IGF-1 (Fig. 5) or even at 10× higher concentrations as tested in our previous work using bovine cartilage explants [13, 57–59].

CPC-IGF-1, therefore, has a high clinical potential and can facilitate the use of IGF-1 for OA therapy with only a single dose, thereby eliminating toxicity concerns. This cationic peptide motif is a versatile carrier that can be used to deliver other protein and peptide-based OA drugs with similar molecular weight as IGF-1 via direct IA injection or delivery of carrier-absorbed therapeutic grafts [60]. Ongoing work includes investigating pharmacokinetics and biodistribution of CPC-IGF-1 conjugate in healthy and arthritic knee joints in vivo as well as evaluating its biological efficacy with a single dose administration in post-traumatic animal models.

## Conclusions

This works shows that by making OA drugs cationic, their cartilage targeting property can be significantly improved. A pro-anabolic growth factor, IGF-1 was electrically charged by conjugating it to cartilage targeting peptide CPC +14N, which provided sufficient electrical driving force to carry IGF-1 into cartilage and created a drug depot that was effective in providing therapeutic doses to chondrocytes over 2 weeks with only a single dose. The therapeutic response from a single dose of CPC-IGF-1 was only matched by the continuous dose of unmodified (free) IGF-1, which is equivalent to multiple IA injections to patients and not clinically feasible. Therefore, CPC-mediated delivery of IGF-1 can enable its clinical use for OA treatment. CPC is a versatile cationic motif that can be used for intra-cartilage delivery of other similar-sized drugs including anti-catabolic agents for combination therapy.

## Data Availability

All data generated or analyzed during this study are included in this published article and its supplementary information files.

## Abbreviations

AA: Ascorbic acid
ACL: Anterior cruciate ligament
CPC: Cationic peptide carrier
DI: Deionized
DMEM: Dulbecco’s modification of Eagle’s medium
DMMB: Dimethyl-methylene blue
DMSO: Dimethylsulfoxide
DZ: Deep zone
ERK: Extracellular signal-regulated kinases
FCD: Fixed charge density
FDA: Fluorescein diacetate
FITC: Fluorescein isothiocyanate isomer 1
GAG: Glycosaminoglycan
HB: Heparin-binding
HEPES: 4-(2-Hydroxyethyl)-1-piperazineethanesulfonic acid
HPLC: High-performance liquid chromatography
IA: Intra-articular
IGF-1: Insulin-like growth factor-1
IGF-1R: Insulin-like growth factor-1 receptor
IGF-BP: Insulin-like growth factor-1 binding protein
IL-1: Interleukin 1
IL-1Ra: Interleukin-1 receptor antagonist
MALDI-TOF: Matrix-assisted laser desorption/ionization time-of-flight
MAPK: Mitogen-activated protein kinases
mAV: Multi-arm Avidin
mTOR: Mammalian target of rapamycin
MW: Molecular weight
MWCO: Molecular weight cut-off
NEAA: Non-essential amino acids
NF-kB: Nuclear factor kappa B
NHS: N-Hydroxysuccinimide
NO: Nitric oxide
OA: Osteoarthritis
PAMAM: Polyamidoamine
PBS: Phosphate-buffered saline
PI: Propidium Iodide
PI3K/Akt: Phosphatidylinositol-3-kinase
PSA: Penicillin streptomycin antibiotic-antimycotic
SF: Synovial fluid
SZ: Superficial zone
TNF-α: Tumor necrosis factor alpha
